# The Influence of the Heat-Affected Zone Mechanical Properties on the Behaviour of the Welding in Transverse Plate-to-Tube Joints

**DOI:** 10.3390/ma11020266

**Published:** 2018-02-09

**Authors:** Miguel Lozano, Miguel A. Serrano, Carlos López-Colina, Fernando L. Gayarre, Jesús Suárez

**Affiliations:** Department of Construction and Manufacturing Engineering, University of Oviedo, 33203 Asturias, Spain; lozanomiguel@uniovi.es (M.L.); lopezpcarlos@uniovi.es (C.L.-C.); gayarre@uniovi.es (F.L.G.); suarezg@uniovi.es (J.S.)

**Keywords:** HAZ mechanical properties, welded coupons, numerical simulation, structural steel joints

## Abstract

Eurocode 3 establishes the component method to analytically characterize the structural joints between beam and columns. When one of the members involved in the joint is a hollow section (i.e., a tube) there is a lack of information for the specific components present in the joint. There are two different ways to bridge the gap: experimental testing on the actual beam column joints involving tubular sections; or numerical modelization, typically by means of finite element analysis. For this second option, it is necessary to know the actual mechanical properties of the material. As long as the joint implies a welding process, there is a concern related to how the mechanical properties in the heat-affected zone (HAZ) influence the behavior of the joint. In this work, some coupons were extracted from the HAZ of the beam-column joint. The coupons were tested and the results were implemented in the numerical model of the joint, in an attempt to bring it closer to the experimental results of the tested joints.

## 1. Introduction

In the steel construction industry, joints are the most critical points in the whole structure. In the case of structural buildings, the most common joint is the beam-column joint. A joint between a horizontal beam that supports vertical loads connected to a column transmitting internal forces through the joint. The European code of design [1] classifies joints as rigid, semi-rigid or simple joints according to the joint stiffness, presenting some boundaries in which the joint and the beam stiffness are compared. The code also classifies the joints according to their moment resistance as full resistance joints, partial resistance joints or simple joints. For this classification, the resistance of the joints is compared with the bending moment resistance of the connected beam. The beam-column joints often behave as semi-rigid, which means the beam transmits bending moment to the column proportionally to the joint stiffness. To correctly evaluate the joint behaviour and to know, as well, the amount of the bending moment transmitted through the joint, it is necessary to characterize it, and then to execute the joint consistently with the provisions of the design.

The Eurocode 3 [1] includes the component method as an approach for the characterization of joints between open profiles, but it does not include a consistent approach when a member of the joint is a hollow section. Nevertheless, the use of hollow sections has increased, because its advantages [2] lead to more effective solutions. Therefore, there are large groups of potentially interesting joints with hollow sections that cannot be characterized because their components have not yet been included in the component method. Some previous research [3,4] has been done to widen the scope of the component method in order to cover the hollow sections. In these references, some analytical equations supported by experimentation and numerical analysis have been presented to be able to characterize some individual components of the joint as a previous step to joint characterization.

**Figure 1:**
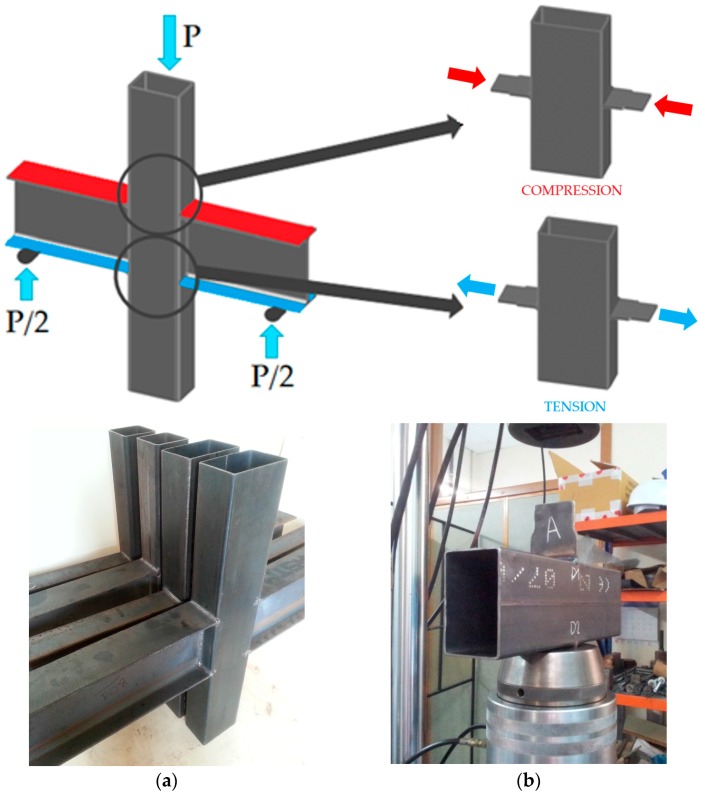
(**a**) Double-sided beam-to-column joints; (**b**) Double-sided lateral plate-to-tube joint.

In an attempt to partially bridge this gap, the authors have been working on a research project focused on the characterization of some components present in a double-side beam-column joint. Figure 1a shows this kind of joint, where the beams are IPE (European Profile I-shaped) open profiles and the column is a rectangular hollow section (RHS) or a square hollow section (SHS). The IPE profile is very efficient for bending on the major axis, which is the common situation. The RHS is one of the most widely used hollow-section columns, allowing simple welded connections due to their flat faces. In the proposed beam-column joint, the flanges and web of beams are welded to the faces of the hollow section. When the beams are under bending their upper flanges transmit transverse compression, and the lower flanges transmit transverse tension to the faces of the tubular column. A simplification of the full beam-column joint is a double-sided welded plate-to-tube joint, see Figure 1b. The transverse plates simulate the upper or the lower flanges of the beam by applying compression or tension loads on both sides of the tube.

As it is a welded joint, another important consideration is the well-known fact that the process of welding affects the material properties in the heat-affected zone (HAZ). Research has been done into the simulation of welded joints involving hollow sections where the finite element method (FEM), validated and calibrated by tests, is used to perform parametric studies. One interesting work is [5], an overview of the main aspects of finite-element analyses relevant to welded hollow-section joints, such as the choice of element type, material non-linearity and modeling of the welds. Some more specific examples are [6,7], where results of experimental tests and corresponding numerical analyses on longitudinal and through plate-to-HSS (hollow-structural-section) welded connections are presented. Some research effort has been devoted [8] to the modeling of damage and residual stresses induced by welding. In [9] the residual stresses are studied during one-pass arc welding in a steel plate. According to this research, the residual stresses clearly decrease in a short distance from the weld bead in case of welds lengths lower than 50 mm and thicknesses in the range of 5 to 8 mm. Other researchers have studied techniques to measure mechanical properties for small-scale pieces as in [10], where residual stresses and elastic moduli are measured, or [11], in which the pillar splitting technique for micro-scale fracture toughness assessment is presented with good correlation between experiments and simulations. In [12], the thermo-mechanical properties in multi-pass welds by means of numerical and experimental approach were quantified. Some of the most accurate characterization methods for the HAZ are:

- Gleeble can be used for the production of microstructure samples and thermomechanical simulation using moving heat source thermal models. The introduction of cooling rates in these models makes the prediction of microstructures possible [13].
- The synchrotron-based X-ray can be used to monitor HAZ microstructural evolution in situ. The temperature-time evolution of the grain-size distribution and micro-strain can be monitored in detail at specimen-relevant scales [14].
- Small punch tests can be used for the mechanical characterization of the different zones that make up the HAZ [15].

This paper is focused on the numerical modeling of the transverse plate-to-tube joint including the actual material properties in the heat-affected zone. The component “lateral faces of a rectangular hollow section” is studied under compression and tension in order to assess their initial stiffness and to propose a new design equation [16] to include joints involving hollow sections in the component method. Initial stiffness is the most important parameter for a joint characterization. However, although there are some equations to determine joint resistance, there are none for joint stiffness.

The mechanical properties of the HAZ have been evaluated by means of tensile tests assisted by digital image correlation (DIC). DIC is used to determine the local displacement gradients and full-field displacements during a uniaxial tension test. The results are implemented in the numerical FE (Finite Element) study of the joint to reach a better validation of the model with respect to the experimental results.

## 2. Experimental Program

The experimental work in this paper has two parts. One is related to the whole procedure that determines the extension and the material properties of the HAZ after the welding process. The second refers to the tests carried out on six double-sided transverse plate-to-tube joints, which are later used to validate and calibrate the numerical model.

### 2.1. Tests Related with the HAZ Properties

Material properties of the HAZ were obtained from tensile specimens extracted from four kinds of welded coupons. Two different configurations for each tube thickness were studied. For each tube thickness, the coupons were welded in two different configurations. The first configuration was a directly opposed twin fillet welded joint, with the dimensions of plate and tube used later in the joint tests and in the FEM calculations. The second configuration was a flat butt welded joint, equivalent to the previous configuration in terms of the thickness of the plates, the three-dimensional heat dissipation, the shrinkage and transformation stresses and the heat input during welding. Twelve specimens were tested with three repetitions for any tube/plate combination and for any of the two described configurations.

**Figure 2:**
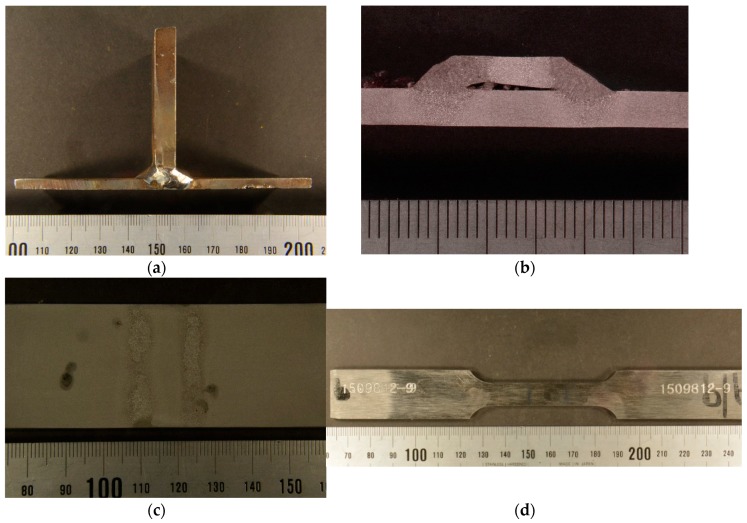
Opposed twin fillet welded joint of 8 mm plate to 4 mm tube test specimens: (**a**) As welded coupon; (**b**) Macrographic examination of the welded coupon shown in figure (**a**); (**c**) Machined and etched welded coupon after 8 mm plate removal; (**d**) Tensile test specimen.

**Figure 3:**
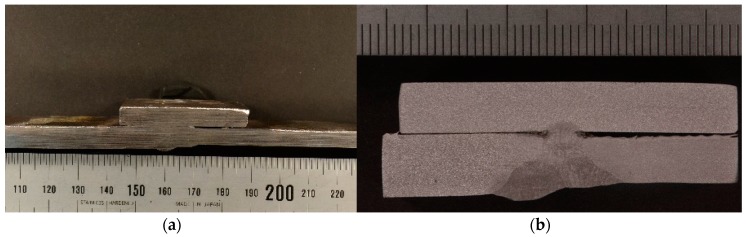
Complete butt penetration welded joint of 8 mm plate to 6 mm tube test specimens. (**a**) As welded coupon; (**b**) Macrographic examination of the welded coupon showed in figure (**a**).

The tension specimens were extracted from welded T-connections and complete butt joint penetration specimens. The specimens were made of 8 mm thick S275 steel plate welded to two structural tubes of 4 and 6 mm thick, respectively. The pieces were welded using electrode arc welding following the welding procedure specifications (WPS). The welding was performed by a manual shielded metal arc welding (SMAW) process. A basic consumable coated electrode AWS/ASME: A5.1; SFA 5.1 E7018 was used. The specimens for the tensile test were then extracted from the base metal, the deposited metal and the HAZ, as shown in Figure 2. In the fillet joint configuration, see Figure 2a, the 8 mm thick plate was removed and the surface of the tube was machined, see Figure 2c. Figure 3 shows a sample for the plate-tube butt-welded joint configuration involving a plate of 8 mm and a tube of 6 mm. 

Tensile tests were performed using an Instron 5583 with a strain rate of 15 MPa/seg using flat-shaped specimens 3 mm thick and 10 mm in section. The length of the narrow portion of the specimen was 10 mm. For each configuration, at least 3 tensile tests were done. During the tensile tests, precise strains were measured by an optical strain gauge system on the basis of digital image processing.

A commercial INSTRON AVE 2 digital image correlation system (Norwood, MA, USA) was used to measure the displacement field during loading. The AVE uses a high-resolution digital camera and advanced real-time image processing to make precise axial and transverse strain measurements.

Each welded coupon was sectioned through areas of interest with a bandsaw followed by a low-speed precision diamond-wafering saw. Four specimens were removed by cutting along the weld bead, retaining some portion of the weldment and base material.

**Figure 4:**
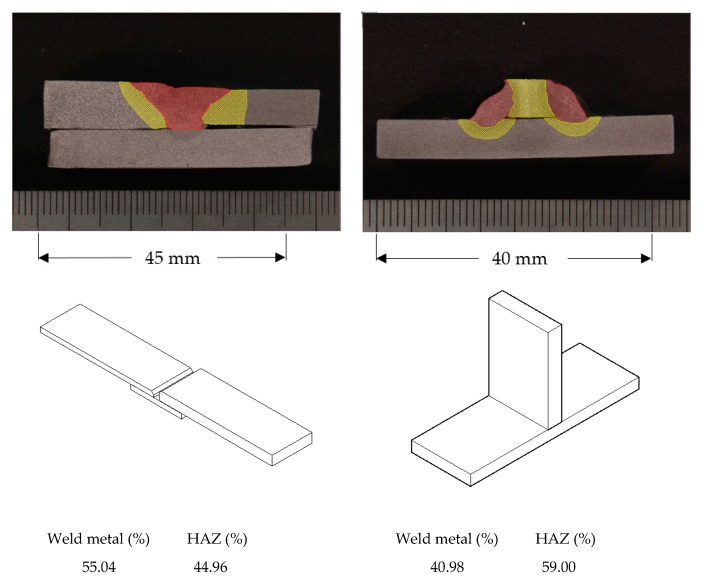
Measurement of the Heat-Affected Zone (HAZ) size and average of relative areas of weld metal and HAZ. (**a**) Complete butt penetration welded joint. (**b**) Opposed twin fillet welded joint.

The samples were subjected to wet grinding and were polished, starting with 180 grit wet paper and followed by 220, 320, 400, 600, and 1200 grit wet paper, 6 micron diamond, and finally 1 micron diamond polishing to finish off the samples. The cross-section samples were etched and examined under an optical microscope. The etchant used was a 2% Nital solution. The results of the tensile tests are presented in Section 4. The area of weld metal and the heat-affected zone (HAZ) was quantified. Figure 4 shows the measured sizes for samples of both configurations, a complete butt penetration joint on the left and an opposed twin fillet welded joint on the right. A sketch of these two configurations, together with the average of the relative areas of the weld metal and the HAZ are also presented in Figure 4.

### 2.2. Tests on Plate-to-Tube Joints

**Figure 5:**
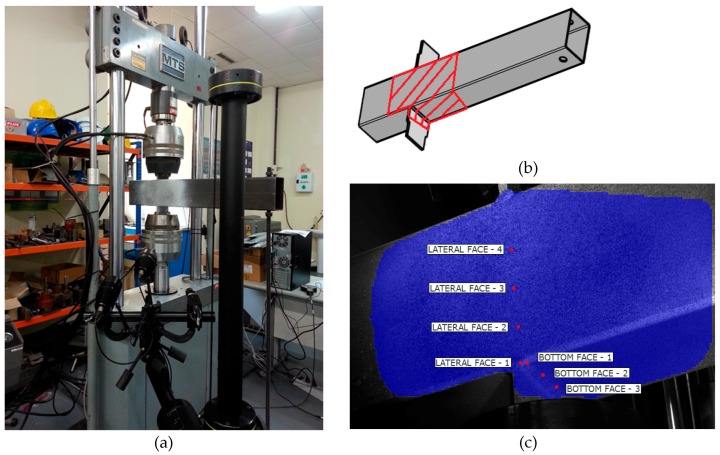
Compression test conducted with the support of Aramis 5M DIC system. (**a**) General set-up. (**b**) Area of interest. (**c**) Reference points.

**Figure 6:**
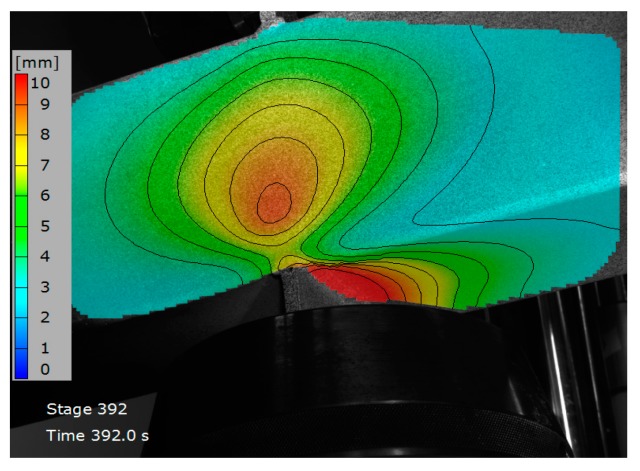
Sample of displacements map.

Some experimental tests were carried out to characterize the transverse plate-to-tube joints shown in Figure 1b. In these tests, a tension or a compression load is transmitted through a pair of plates welded to the tube in a transversal position, simulating the flanges of the open profile beam. Subsequently, the pieces are positioned in a universal testing machine, MTS with 250 kN of load capacity. In order to obtain force-displacement plots on several points of the rectangular hollow section, tension or compression loads were applied to the plates until the maximum load was reached. For the tests on joints under compression, a rod was positioned at one end of the tube to prevent rotation. The tests were carried out under displacement control at a speed of 2 mm/min. Aramis 5M 3D DIC equipment (GOM GmbH, Braunschweig, Germany) was used to measure the deformations during the tests more accurately. This measuring technique is non-contact, material-independent and provides accurate 3D surface coordinates, 3D displacements, surface strain values and strain rates. By means of this system, the displacements synchronized with the applied load are obtained over a large area on which the cameras are focused. Figure 5 shows a joint during a compression test. The area of interest is marked on the specimen and a third picture shows the chosen reference points on the lateral wall and on the bottom face of the tubular section. Figure 6 shows a map of curves with the same displacement.

From an extensive experimental program carried out on these joints, six of them (three in tension and three in compression) were selected to validate the numerical model. These specific joints combined tubes of RHS200 × 100 × 6, RHS150 × 100 × 4 and RHS100 × 100 × 6 with plates of 8 mm thick. Results of these tests selected for comparison with the FEA are presented in Section 4.

## 3. Numerical Simulation

The transverse plate-RHS joint was modeled in the general purpose finite element software ANSYS 16.1 (16.1, ANSYS, Inc., Canonsburg, PA, USA), including material and geometrical non-linearities. Solid-186 elements were selected for these simulations. At least three solid elements were placed through the thickness, following one of the most generalized rules of thumb for accurately obtain bending stresses. The authors have previously used four elements through the thickness for this type of simulation of transverse compression in rectangular hollow sections [4], but the deeper experimental validation and calibration of the mesh size in plate-tube welded joints presented later in [17] lead to simplifying the mesh by including a minimum number of three elements in the wall thickness with sizes between 3 and 5 mm, for the area closest to the connection and the rest of the model, respectively. The number of elements varied from 15,700 to 22,900 in the six finite element models, and the number of nodes from approximately 59,500 to 87,800. The solid-186 element, which is initially a 20-node hexahedral element, is automatically transformed by ANSYS into different shapes to model geometrical and mesh transitions. This caused approximately the 85% of the meshes to be created with the mentioned hexahedrons, but the rest were 13-node pyramids (8%), 15-node prisms (3.5%) and 10-node tetrahedrons (3.5%). The model was loaded by applying a controlled displacement, and a double symmetry was considered in order to save computational time.

**Figure 7:**
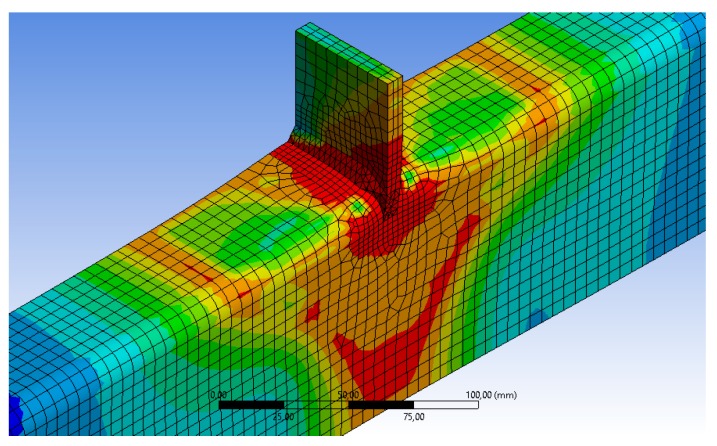
FEA model.

The welds were geometrically idealized as a conjunction of prismatic and tetrahedral parts, as can be seen in Figure 7. However, the real measured dimensions of the weld beads were used in the model. For the material model of the weld bead, the nominal values provided by the manufacturer of the electrodes were used (*f_y_* = 500 MPa, *f_u_* = 570 MPa). Since the results obtained from tests in Section 2.1 did not give worse values of yield and ultimate strength of the material for the HAZ than for the original material of the RHS and the loading plate (see Table 1), the HAZ was not considered as a different part. However, a previous work [18] suggested that in cold-formed RHS, it is necessary to consider the strain hardening caused mainly at the corners during the forming process. In order to take this into account, the present FE model uses the Eurocode proposal for the average material properties of cold-formed open profiles. The code gives an average yield limit *f_ya_* from the yield and ultimate strength of the base material (*f_yb_*, *f_ub_*), and takes into account the thickness (*t*) and the cross-section area (*A*). See Equation (1):

(1) $$f_{ya}=f_{yb}+\left(28\times \frac{t^{2}}{A}\right)\times \left(f_{ub}-f_{yb}\right)$$

Since the base properties of the material are not known, the properties of the material of the faces are used instead. A bi-linear material model was used in the FEM by taking the elastic part up to the yield limit and then, an approximated tangent modulus of 1000 MPa.

## 4. Results and Discussion

**Figure 8:**
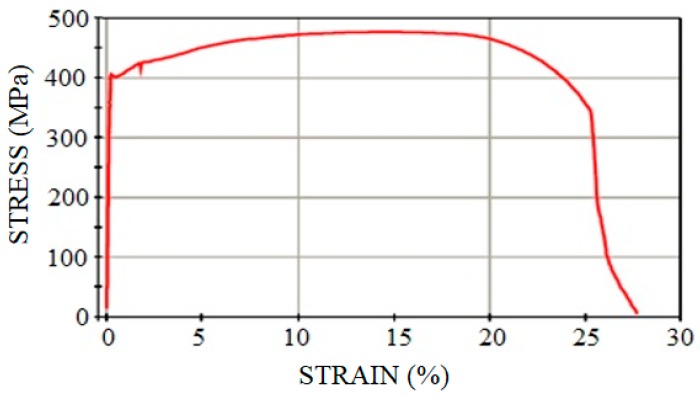
Stress-strain curve for a coupon from plate 8 mm + tube 4 mm.

**Figure 9:**
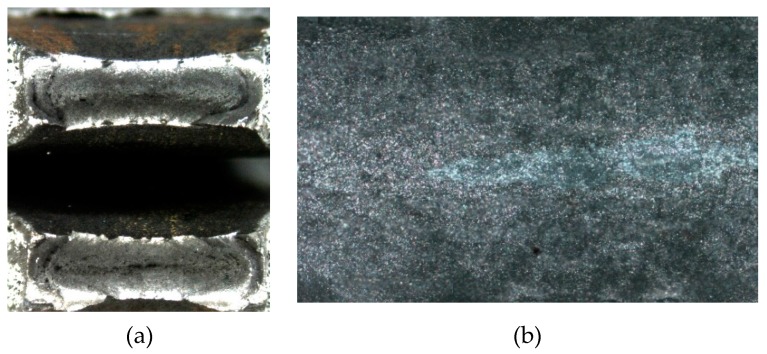
Fracture cup-cone area (**a**). Fracture surface with NIKON EPIPHOT 200 (**b**).

Table 1 shows the actual mechanical properties of the tubes and plates of the non-affected material and of the HAZ material. The yield limit *f_y_*, the ultimate strength *f_u_* and the Young´s modulus, *E*, for both zones are presented. The samples tested and the results shown in the non-affected material column of Table 1 were obtained before the welding process, following the same procedure described in Section 2.1 for the HAZ area. The fracture of coupons was always ductile with a well-developed plateau after the yield point. Figure 8 shows, as an example, the stress-strain curve plotted during the standard tensile test for one coupon, combining a plate of 8 mm with a tube thickness of 4 mm. Figure 9 shows, on the left, an image taken in the fracture cup-cone area with a stereoscope magnifier NIKON SMZ800; and on the right, it is possible to see the fracture surface in an image captured by an optical microscope NIKON EPIPHOT 200.

As the results of the base material are not significantly different from the HAZ, we conclude that there is not a clear deterioration of these materials after welding.

The main objective of this work was to assess the initial stiffness of the component “lateral faces of RHS” in order to obtain a new design equation [16] to allow the component method to be used for joints with hollow sections. The initial part of the force-displacement curve was studied, measuring the relative displacement between the center of the welded joint and the horizontal mid-plane of the RHS. Measurements were taken both in experimental tests and in the finite element models.

**Figure 10:**
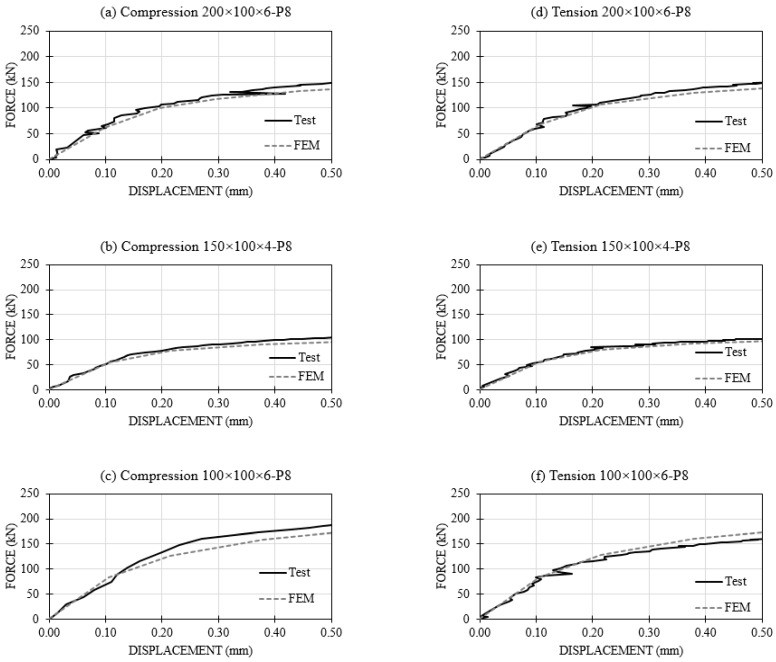
Test vs. FEM in load-displacement curve. (**a**) Compression 200 × 100 × 6-P8; (**b**) Compression 150 × 100 × 4-P8; (**c**) Compression 100 × 100 × 6-P8; (**d**) Tension 200 × 100 × 6-P8; (**e**) Tension 150 × 100 × 4-P8; (**f**) Tension 100 × 100 × 6-P8.

In Figure 10, the force-displacement curves up to a displacement of 0.5 mm are shown. This value is half of 1% of the face width, which is generally taken as the serviceability deformation limit in joints involving RHS [19]. A good agreement can be observed between experimental tests and the FE model.

Table 2 presents the initial stiffness obtained in tests and with the FE models for the different samples and simulations and differences between them. This table shows that a good agreement was reached for this sensitive property, thus validating the numerical models.

The oxidation effects for welds of similar characteristics to those used in this work and carried out with the same type of electrode E7018 and welding process SMAW were studied in [20]. The authors concluded that the amount of deposited manganese metal and silicon led to a reduction of the oxygen present, decreasing the effects of a potential oxidation. An increase in hardness and ultimate strength was also observed.

With regard to the metallographic analysis, in [21], the authors studied the composition and properties of the weld metal for an electrode E7018, such as the one used in the present work. After several tests the authors reported that the microstructure of a weld bead deposited with this type of electrode was mainly composed of acicular ferrite, grain boundary ferrite and second-phase aligned ferrite. They also confirmed that when welding with this type of electrode, the deposited metal manganese and silicon was high.

## 5. Conclusions

An examination of the initial stiffness of the component “lateral faces of RHS” in transverse plate-to-tube joints, suggests that it may be possible to extend the component method to joints involving hollow sections. The examination involved a numerical finite element parametric study considering the joint under both tension and compression. The actual material properties of the heat-affected zone, as well as the extension of the HAZ, were obtained. These results make it possible to decide whether the numerical model should include the HAZ as a differentiated area. From this research, some observations and conclusions can be outlined.

First, the material properties of the HAZ of steel were not deteriorated in the welding process.

Second, the numerical model to simulate the joints provides very good agreement with the test results from the plate-to-tube joints in terms of the initial stiffness.

Third, the parametric model developed in this paper is a cost-effective tool for assessing the component “lateral faces” of a rectangular hollow section when it is connected to a transverse plate.

Future work will be focused on the extension of the model to consider the full beam-column joint described in this paper, and on proposing an analytical equation to evaluate the stiffness of the component.

## Figures and Tables

**Table 1:** Actual material properties in base material and HAZ.

RHS Tube or Plate	Non-Affected Material			HAZ Material		
	*f_y_*	*f_u_*	*E*	*f_y_*	*f_u_*	*E*
	(MPa)	(MPa)	(GPa)	(MPa)	(MPa)	(GPa)
RHS *t* = 4 mm	369	459	195	407	477	197
RHS *t* = 6 mm	433	503	201	446	561	204
Plate of 8 with tube of 4 mm	364	516	213	365	494	212
Plate of 8 with tube of 6 mm				381	492	224

**Table 2:** Comparison of initial stiffness in joints.

Tube	Stiffness TEST (kN/mm)		Stiffness FEM (kN/mm)		Difference (%)	
	Tension	Compression	Tension	Compression	Tension	Compression
RHS 150 × 100 × 4-P8	517.1	508.8	506.3	512.5	−2.1	0.7
RHS 200 × 100 × 6-P8	604.6	606.8	615.9	615.8	1.9	1.5
RHS 100 × 100 × 6-P8	737.2	727.5	756.8	752.5	2.7	3.4
